# Comparing Mortality Risk Predictive Ability of Different Scoring Systems in Cirrhotic Patients with Bacteremia

**DOI:** 10.1155/2020/8596567

**Published:** 2020-10-26

**Authors:** Chi-Chieh Hung, Yin-Chou Hsu, Kuo-Hsuan Lin

**Affiliations:** ^1^Department of Emergency Medicine, E-Da Hospital, I-Shou University, Kaohsiung, Taiwan; ^2^School of Medicine, College of Medicine, I-Shou University, Kaohsiung, Taiwan

## Abstract

Patients with liver cirrhosis and bacteremia have substantially higher risk of mortality and morbidity. Our study aimed to investigate scoring systems that can predict the mortality risk in patients with cirrhosis and bacteremia. A single-center, retrospective cohort study was performed among adult patients who visited the emergency department from January 2015 to December 2018. All patients diagnosed with liver cirrhosis and bacteremia were enrolled and divided into survivor and nonsurvivor groups for comparison based on their 30-day in-hospital mortality event. The Pitt bacteremia score (PBS), model for end-stage liver disease (MELD) score, Child–Pugh score, and quick sequential Organ Failure Assessment (qSOFA) score were calculated and compared using the area under the receiver operating characteristic (AUROC) curves. A total of 127 patients (survivor: 86; nonsurvivor: 41) were eligible for this study. Compared with the nonsurvivor group, patients in the survivor group had significantly lower MELD score (22 ± 7 vs. 29 ± 5, *p* < 0.001), lower proportion of high qSOFA (score ≥ 2) (23.3% vs. 51.2%, *p* < 0.01), and high PBS (score ≥ 4) (7.0% vs. 34.1%, *p* < 0.001) category. There was also a significantly different distribution in Child–Pugh classification between the two groups (*p* < 0.01). The survivor group had significantly lower proportion of acute-on-chronic liver failure (27.9% vs. 68.3%, *p* < 0.001) and fewer number of organ failures (*p* < 0.001). In comparison of the discriminative ability in mortality risk prediction, PBS (AUROC = 0.83, 95% CI = 0.75–0.90, *p* < 0.001) and MELD scores (AUROC = 0.78, 95% CI = 0.70–0.86, *p* < 0.001) revealed a better predictive ability than Child–Pugh (AUROC = 0.69, 95% CI = 0.59–0.70, *p* < 0.01) and qSOFA scores (AUROC = 0.65, 95% CI = 0.54–0.75, *p* < 0.01). PBS and MELD scores both demonstrated a superior ability of predicting mortality risk in cirrhotic patients with bacteremia.

## 1. Introduction

Liver cirrhosis is the 14th most common cause of death worldwide [1], and cirrhosis-related mortality has continued to increase in the recent decades [2]. Altered intestinal motility, bacterial overgrowth, increased intestinal permeability, and cirrhosis-associated immune dysfunction all predispose cirrhotic patients to infection [3]. The incidence of infection in cirrhotic patients increases as their liver function worsens; and mortality risk was four times higher than for those without cirrhosis [1].

Bacteremia (i.e., bloodstream infection) is a severe and life-threatening condition that requires prompt and aggressive treatment [4]. Liver cirrhosis is a risk factor associated with death in patients with bacteremia, with a 30-day mortality rate ranging from 39.4–54.8% [5]. The Pitt bacteremia score (PBS) is a widely used scoring system to determine the prognosis and mortality risk for patients with bacteremia [6]; it has not been validated for patients with cirrhosis. Similarly, while the Child–Pugh score and the model of end-stage liver disease (MELD) score are the most frequently used scoring systems to assess the severity and prognostic significance in patients with end-stage liver disease [7, 8], they have not been validated for cirrhotic patients with bacteremia. This study aimed to compare the mortality prediction ability of different scoring systems for cirrhotic patients with bacteremia. We also incorporated the quick sequential Organ Failure Assessment (qSOFA) score for comparison, which was suggested by a recent clinical practice guideline for management of patients with decompensated cirrhosis [9].

## 2. Methods

### 2.1. Study Design

We conducted a retrospective observational cohort study at a tertiary referral medical center located in southern Taiwan with approximately 57,000 emergency department (ED) visits per year. All adult patients (aged ≥18 years) who visited the ED from January 1, 2015, to December 31, 2018, and fulfilled both criteria were enrolled. Those criteria were positive blood culture results during hospitalization and the ED diagnostic code for liver cirrhosis. An episode of bacteremia was defined as the presence of microorganisms in the blood in either of the two situations: blood culture of ≥2 sets collected from separate sites yielding the same pathogen or 1 set of blood culture yielding bacteria compatible with the patient's clinical manifestations. The diagnosis of cirrhosis was confirmed by liver biopsy results or a combination of imaging and clinical results of each patient. Patients with fungemia, contaminated blood samples, prior antibiotics use, or no progression to cirrhosis were excluded. This study was carried out in accordance with the principles of the Declaration of Helsinki, and the protocol was approved by the local Institutional Review Board. The requirement for informed consent was waived due to the retrospective observational nature of the study.

### 2.2. Data Collection

All eligible patients were divided into survivor and nonsurvivor groups for further analysis. We collected their baseline characteristics, comorbidities, prior medications, laboratory results, source of bacteremia, and microbiological data from manual chart review and electronic medical records. The etiology of liver cirrhosis was classified as alcohol, viral hepatitis (e.g., Hepatitis B or C), or other causes (e.g., cryptogenic cirrhosis). Cirrhotic patients were stratified according to their Child–Pugh scores and MELD scores [7]. The source of bacteremia was classified as respiratory tract infection (radiological increased infiltration combined with clinical symptoms), urinary tract infection, spontaneous bacterial peritonitis (diagnostic paracentesis with a polymorphonuclear leukocyte count of ≥250 cells/*μ*L), biliary tract infection, soft tissue infection, and primary bacteremia (source of unknown origin).

### 2.3. Definitions

The presence of acute-on-chronic liver failure (ACLF) and calculation of the chronic liver failure consortium organ failure (CLIF-C OF) score including the number of organ failure events was defined according to the CANONIC study [8]. The qSOFA score was calculated using the initial ED triage parameter: the Glasgow coma score <15, respiratory rate ≥22 breaths/min, and systolic blood pressure ≤100 mmHg [10]. A qSOFA score of ≥2 points was used as the prognostic cutoff value based on previous studies [11]. PBS was calculated for each patient at the onset of bacteremia based on five variables: blood pressure, temperature, requirement of mechanical ventilation, recent cardiac arrest, and mental status [6]. The PBS ≥4 was used as an indicator for poor prognosis and risk of death [12]. The Systemic Inflammatory Response Syndrome (SIRS) criteria were defined according to the 2012 Surviving Sepsis Campaign [13]. The patients were empirically treated with antibiotics in accordance with the recommendations of the European Association for the Study of Liver [14] and local epidemiological data (e.g., amoxicillin/clavulanic acid for soft tissue infection, ceftriaxone for spontaneous bacterial peritonitis, and moxifloxacin for respiratory tract infection). The administration of antibiotics was considered appropriate if the cultured pathogen was susceptible based on the results of an *in vitro* susceptibility test.

### 2.4. Outcome Measurement and Statistical Analysis

The primary outcome of this study was 30-day in-hospital mortality. Data were presented as mean with standard deviation or medians, with interquartile range for continuous variables and numbers (%) for categorical variables. The differences between the survivor and nonsurvivor group were compared using the paired *t*-test and chi-square test (or Fisher's exact test) for the continuous and categorical variables, respectively. The Mann–Whitney test was used for continuous variables if data were not normally distributed. The scoring systems included in our study were tested for the discriminative ability in predicting the risk of mortality in cirrhotic patients with bacteremia using the area under the receiver operating characteristic (AUROC) curves. A two-tailed *p* value <0.05 was considered statistically significant. All statistical analyses were performed using the Statistical Package for the Social Sciences version 22.0 (SPSS Inc., Chicago, IL, USA) and MedCalc version 18.2.1 software.

## 3. Results

During the study period, a total of 252384 patients visited the ED; among them, 158 patients met the selection criteria. After excluding patients with fungemia (*N*  = 3), contaminated blood samples (*N*  = 11), prior antibiotics use (*N*  = 6), and no progression to cirrhosis (*N*  = 11), the remaining 127 patients were finally analyzed.

As shown in Table 1, the overall 30-day in-hospital mortality rate of our patients was 32.3% (41/127). The mean age was 56.1 ± 12.5 years, and 77.2% (98/127) were male patients. More than half (52.8%) of the patients developed cirrhosis attributed to alcohol use; and the majority of them had advanced stage of cirrhosis based on MELD scores and the Child–Pugh classification. In sepsis severity stratification (Table 2), most of them (73.2%) met the SIRS criteria, whereas a small proportion of patients belonged to the high qSOFA (32.3%) and high PBS (15.7%) group according to the definitions. A total of 52 patients (40.9%) had ACLF, and more than half (64.6%) of the patients had organ failure event according to the CLIF-C OF score. Regarding the source of bacteremia, spontaneous bacterial peritonitis (34.6%) accounted for the leading cause of infection, followed by biliary tract (21.3%) and soft tissue (16.5%) infection. As for the causative microorganism in patients with cirrhosis, the Gram-negative strains were the predominant pathogens (57.5%).

In the subgroup analysis between the survivor and nonsurvivor group, there were no significant differences in age, gender, cirrhosis etiology, comorbidities, and microbiological distribution. It was notable that compared with the nonsurvivor group, the patients in the survivor group had significantly lower MELD score (22 ± 7 vs. 29 ± 5, *p* < 0.001); also, a significantly different distribution in Child–Pugh classification between the two groups was demonstrated (*p* < 0.01). Regarding the laboratory results, patients in the survivor group had significantly lower serum bilirubin (3.9 mg/dL vs. 6.3 mg/dL, *p* < 0.01), creatinine (1.3 mg/dL vs. 1.8 mg/dL, *p* < 0.01), and international normalized ratio (INR) level (1.4 vs. 1.8, *p* < 0.001). In sepsis severity stratification (Table 2), patients in the survivor group had a significantly lower proportion (27.9% vs. 68.3%, *p* < 0.001) of ACLF developed, as well as a lesser number of organ failure events (*p* < 0.001). The survivor group had a significantly lower mean CLIF-C OF score (8 ± 1 vs. 11 ± 2, *p* < 0.001); also, it had fewer patients classified to high qSOFA (23.3% vs. 51.2%, *p* < 0.01) and high PBS (7.0% vs. 34.1%, *p* < 0.001) categories. The distribution of the causative microorganism and proportion of appropriate antibiotics administration were similar in both groups (Table 2).

**Figure 1:**
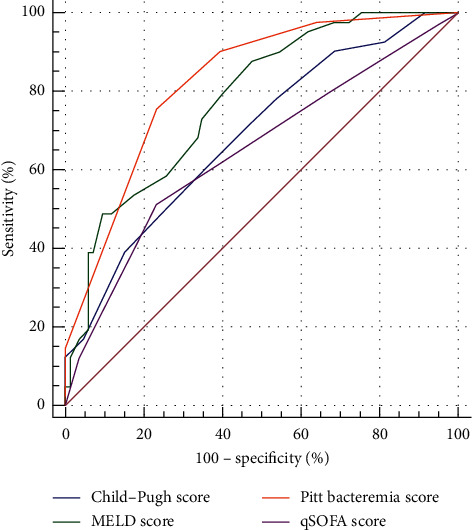
Receiver operating characteristic curves for 30-day mortality predicting ability of four different scoring systems in cirrhotic patients with bacteremia.

We compared the discriminative ability of four different scoring systems in predicting the risk of mortality in cirrhotic patients with bacteremia using AUROC curves. As shown in Figure 1, the AUROC of each scoring system was as follows: PBS, 0.83 (95% confidence interval (CI) = 0.75–0.90, *p* < 0.001), MELD, 0.78 (95% CI = 0.70–0.86, *p* < 0.001), Child–Pugh, 0.69 (95% CI = 0.59–0.79, *p* < 0.01), and qSOFA, 0.65 (95% CI = 0.54–0.75, *p* < 0.01). Furthermore, the AUROC of PBS was significantly higher than the Child–Pugh (*p*=0.01) and qSOFA (*p* < 0.001) scores, and the AUROC of MELD score was significantly higher than the qSOFA (*p*=0.02) score. There was no significant difference between the AUROC of the PBS and MELD scores (*p*=0.36).

## 4. Discussion

In this ED-based single-center retrospective study, we demonstrated the accuracy and ability of PBS and MELD scores to predict the 30-day in-hospital mortality risk in cirrhotic patients with bacteremia. Moreover, as age, gender, infection source, and causative microorganism distribution were revealed to be similar between the survivor and nonsurvivor groups, the significantly poorer renal function, liver function, coagulation function, and cirrhotic stage of the patients in the nonsurvivor group indicated that the severity of the cirrhosis was the predominant cause of mortality in this study. This result further strengthened the importance of host factors in determining infection outcomes [15].

Patients with liver cirrhosis frequently have serious bacterial infections because of their immune system impairment, including decreased complement system synthesis, impaired neutrophil function, and lower number of hepatic Kupffer's cells, which causes blunt inflammatory response, that hinders localization of the primary focus of the infections [15, 16]. Cirrhotic patients with severe bacterial infections may lack the presence of tachycardia, leukocytosis, high fever, or other conventional inflammatory parameters elevation because of their drug use (e.g., beta-blockers) and hypersplenism. Such nonspecific clinical symptoms further lower the warning level of clinicians and result in delayed treatment [17].

Early recognition of infection severity in cirrhotic patients is essential, since serial measures such as prompt broad-spectrum antimicrobial agent administration are crucial for treating cirrhotic patients with sepsis and septic shock [17, 18]. The PBS was originally developed for mortality prediction in patients with *Pseudomonas aeruginosa* bacteremia [6]. PBS has also been validated in other Gram-negative and Gram-positive strains, as well as in nonbacteremic infections, according to a recent prospective study [19]. The simplicity of PBS could identify patients with life-threatening infections based solely on several patient-oriented variables without the need of laboratory measurement [20].

It has been proposed that PBS may have insufficient predictive ability in patients with cirrhosis because fever, hypotension, and altered mental status could be present in cirrhotic patients without infection. However, no study to date has validated these hypotheses [21]. Our study revealed that a solid prognostic correlation between the PBS and mortality outcome in cirrhotic patients with bacteremia could be a valuable finding that warrants confirmation by a larger prospective study in the future.

Compared with other scoring systems, qSOFA unexpectedly revealed a poorer prognostic correlation in this study. According to recent practice guidelines, both Sepsis-3 and qSOFA criteria should be obtained from patients with cirrhosis and bacterial infection if baseline SOFA scores are not readily available [9]. It has been proposed that qSOFA is to perform better than traditional SIRS criteria for severity stratification in cirrhotic patients with bacterial infection. This is because the organ dysfunction parameter has been validated as an important mortality predictor for patients with cirrhosis [8, 18]. In the present study, only cirrhotic patients with bacteremia were enrolled, and they may not be representative of all patients with bacterial infection. This potentially resulted in selection bias and a subsequent failure to demonstrate the prediction accuracy of the qSOFA score for this study.

Child–Pugh and MELD scores have been widely used for prognostic prediction in cirrhotic patients in various conditions. A large number of studies have been conducted to compare the discriminative ability between the two scores; the studies have revealed conflicting results [22]. In patients with cirrhosis and sepsis, the Child–Pugh score had better in-hospital mortality than the MELD score, according to a retrospective study [23]. Another study revealed opposite results [24]. The superior prognostic stratification ability of the MELD score in the present study could provide clinicians the ability to differentiate the mortality risk more rapidly. Clinicians would be able to calculate mortality risk based on laboratory results. Child–Pugh would not be as applicable because its two components, ascites and hepatic encephalopathy, may be influenced by drug use and clinicians' judgment [22].

There were several limitations in the present study, including its retrospective and monocentric nature. First, we did not consider and analyze the timing of antimicrobial administration in our patients, which is crucial for treating patients with cirrhosis and bacterial infection [18]. Second, all data obtained from the manual chart reviews and electronic medical records led to inevitable recall and selection bias. Lastly, the relatively small sample size of the study may limit its extrapolative ability to other facilities.

## 5. Conclusions

This study showed the predictive ability of PBS and MELD scores in 30-day in-hospital mortality risk stratification in patients with cirrhosis and bacteremia. Clinicians can utilize these simple scores more liberally while treating patients with cirrhosis and bacteremia. This will aid in earlier recognition of patients with a high risk of mortality and more prompt treatment.

## Figures and Tables

**Table 1:** Baseline characteristics of cirrhotic patients with bacteremia (*n* = 127).

Characteristics	All (*n* = 127)	Nonsurvivors (*n* = 41)	Survivors (*n* = 86)	*p* value
Age, *y*, mean ± SD	56.1 ± 12.5	56.1 ± 13.1	56.1 ± 12.3	1.00

Male, *n* (%)	98 (77.2)	32 (78.0)	66 (76.7)	1.00

Etiology of cirrhosis, *n* (%)				
Alcohol	67 (52.8)	27 (65.9)	40 (46.5)	0.06
Viral hepatitis	45 (35.4)	10 (24.4)	35 (40.7)	0.08
Other	15 (11.8)	4 (9.8)	11 (12.8)	0.77

Child–Pugh classification, *n* (%)				<0.01^*∗*^
A	7 (5.5)	0 (0)	7 (8.1)	
B	42 (33.1)	9 (22.0)	33 (38.4)	
C	78 (61.4)	32 (78.0)	46 (53.5)	

MELD score, mean ± SD	25 ± 7	29 ± 5	22 ± 7	<0.001^*∗*^

Comorbidities, *n* (%)				
Diabetes mellitus	49 (38.6)	11 (26.8)	38 (44.2)	0.08
Hypertension	45 (35.4)	12 (29.3)	33 (38.4)	0.43
Malignancy	35 (27.6)	13 (31.7)	22 (25.6)	0.53

Laboratory results				
Hemoglobin (g/dL), mean ± SD	10.1 ± 2.3	9.9 ± 2.6	10.2 ± 2.1	0.45
Leukocyte (×10^9^/L), median (IQR)	10.1 (5.5–16.2)	12.5 (4.6–19.7)	9.6 (5.7–13.9)	0.25
Platelet (×10^9^/L), median (IQR)	76 (44–120)	56 (40–124)	82 (53–121)	0.22
INR, median (IQR)	1.5 (1.3–1.8)	1.8 (1.4–2.2)	1.4(1.3–1.7)	<0.001^*∗*^
Bilirubin (mg/dL), median (IQR)	4.9 (2.3–8.5)	6.3 (3.4–13.0)	3.9 (2.1–7.3)	<0.01^*∗*^
Sodium (mmol/L), mean ± SD	130 ± 7	130 ± 7	129 ± 9	0.65
Creatinine (mg/dL), median (IQR)	1.4 (1.0–2.2)	1.8 (1.3–3.5)	1.3 (1.0–1.8)	<0.01^*∗*^
CRP (mg/dL), median (IQR)	32.4 (9.1–91.6)	29.1 (9.9–103.3)	34 (7.9–86.2)	0.45

^*∗*^ *p* < 0.05, SD: standard deviation. MELD: model for end-stage liver disease. IQR: interquartile range. INR: International Normalized Ratio. CRP: c-reactive protein.

**Table 2:** Microbiological distribution, infection source, sepsis severity, and antimicrobial agent administration of cirrhotic patients with bacteremia (*n* = 127).

Characteristics	All (*n* = 127)	Nonsurvivors (*n* = 41)	Survivors (*n* = 86)	*p* value
SIRS, *n* (%)	93 (73.2)	32 (78.0)	61 (70.9)	0.52

qSOFA score, *n* (%)				<0.01^*∗*^
<2	86 (67.7)	20 (48.8)	66 (76.7)	
≥2	41 (32.3)	21 (51.2)	20 (23.3)	

Pitt bacteremia score, *n* (%)				<0.001^*∗*^
<4	107 (84.3)	27 (65.9)	80 (93.0)	
≥4	20 (15.7)	14 (34.1)	6 (7.0)	

ACLF, *n* (%)	52 (40.9)	28 (68.3)	24 (27.9)	<0.001^*∗*^

Number of organ failures, *n* (%)				<0.001^*∗*^
0	45 (35.4)	3 (7.3)	42 (48.8)	
1	33 (26.0)	7 (17.0)	26 (30.2)	
2	38 (29.9)	20 (48.8)	18 (20.9)	
≥3	11 (8.7)	11 (26.8)	0 (0)	

CLIF-C OF score, mean ± SD	9 ± 2	11 ± 2	8 ± 1	<0.001^*∗*^

Infection source, *n* (%)				
Respiratory tract infection	8 (6.3)	4 (9.8)	4 (4.7)	0.27
Urinary tract infection	17 (13.4)	2 (4.9)	15 (17.4)	0.06
Spontaneous bacterial peritonitis	44 (34.6)	19 (46.3)	25 (29.1)	0.07
Biliary tract infection	27 (21.3)	10 (24.4)	17 (19.8)	0.64
Soft tissue infection	21 (16.5)	6 (14.6)	15 (17.4)	0.80
Primary bacteremia	10 (7.9)	0 (0)	10 (11.6)	0.03^*∗*^

Microbiological distribution, *n* (%)				
Gram-positive pathogen	44 (34.6)	13 (31.7)	31 (36.0)	0.69
*Staphylococcus aureus*	14 (11.0)	6 (14.6)	8 (9.3)	
*Streptococcus pneumoniae*	14 (11.0)	4 (9.8)	10 (11.6)	
Viridans Streptococcus	5 (3.9)	1 (2.4)	4 (4.7)	
Others	11 (8.7)	2 (4.9)	9 (10.5)	

Gram-negative pathogen	73 (57.5)	27 (65.9)	46 (53.5)	0.25
*Escherichia coli*	27 (21.3)	11 (26.8)	16 (18.6)	
*Klebsiella pneumoniae*	19 (15.0)	5 (12.2)	14 (16.3)	
*Pseudomonas aeruginosa*	8 (6.3)	4 (9.8)	4 (4.7)	
Others	19 (15.0)	7 (17.0)	12 (14.0)	

Polymicrobial	10 (7.9)	1 (2.4)	9 (10.5)	0.17

Antimicrobial appropriateness, *n* (%)	92 (72.4)	27 (65.9)	65 (75.6)	0.65

^*∗*^ *p* < 0.05 SIRS: systemic inflammatory response syndrome. qSOFA: quick sequential organ failure assessment. ACLF: acute-on-chronic liver failure. CLIF-C OF: chronic liver failure consortium organ failure score.

## Data Availability

The datasets used and/or analyzed during the present study are available from the corresponding author on reasonable request.
